# Relación entre trastornos alimentarios y familia e ideación suicida en adolescentes escolarizadas de Bogotá

**DOI:** 10.15446/rsap.V25n4.97129

**Published:** 2023-07-01

**Authors:** Juan C. González-Quiñones, Sandra P. Morales-Méndez, Sandra P. Calderón-Gutiérrez, Yudy M. Espinosa-Tafur, Santiago J. Arias-Torres, Diana F. Cely Ortegón, Vanessa E. Pinilla-Fonseca, Jenny A. Pinzón

**Affiliations:** 1 JG: MD. M. Sc. Salud Pública. Facultad de Medicina, Fundación Universitaria Juan N Corpas. Bogotá, Colombia. juan.gonzalez@juanncorpas.edu.co Fundación Universitaria Juan N Corpas Facultad de Medicina Fundación Universitaria Juan N Corpas Bogotá Colombia juan.gonzalez@juanncorpas.edu.co; 2 SM: MD. Facultad de Medicina, Fundación Universitaria Juan N Corpas. Bogotá, Colombia. sandra-morales@juanncorpas.edu.co Fundación Universitaria Juan N Corpas Facultad de Medicina Fundación Universitaria Juan N Corpas Bogotá Colombia sandra-morales@juanncorpas.edu.co; 3 SC: MD. Facultad de Medicina, Fundación Universitaria Juan N Corpas. Bogotá, Colombia. sandra-calderon@juanncorpas.edu.co Fundación Universitaria Juan N Corpas Facultad de Medicina Fundación Universitaria Juan N Corpas Bogotá Colombia sandra-calderon@juanncorpas.edu.co; 4 YE: MD. Facultad de Medicina, Fundación Universitaria Juan N Corpas. Bogotá, Colombia. yudy-espinosa@juanncorpas.edu.co Fundación Universitaria Juan N Corpas Facultad de Medicina Fundación Universitaria Juan N Corpas Bogotá Colombia yudy-espinosa@juanncorpas.edu.co; 5 SA: MD. Facultad de Medicina, Fundación Universitaria Juan N Corpas. Bogotá, Colombia. santiago-arias@juanncorpas.edu.co Fundación Universitaria Juan N Corpas Facultad de Medicina Fundación Universitaria Juan N Corpas Bogotá Colombia santiago-arias@juanncorpas.edu.co; 6 DC: MD. Facultad de Medicina, Fundación Universitaria Juan N Corpas. Bogotá, Colombia. diana-cely@juanncorpas.edu.co Fundación Universitaria Juan N Corpas Facultad de Medicina Fundación Universitaria Juan N Corpas Bogotá Colombia diana-cely@juanncorpas.edu.co; 7 VP: MD. Facultad de Medicina, Fundación Universitaria Juan N Corpas. Bogotá, Colombia. vanessa-pinilla@juanncorpas.edu.co Fundación Universitaria Juan N Corpas Facultad de Medicina Fundación Universitaria Juan N Corpas Bogotá Colombia vanessa-pinilla@juanncorpas.edu.co; 8 YP: Ing. Sistemas. Fundación Universitaria Juan N Corpas. Bogotá, Colombia. jenny.pinzon@juanncorpas.edu.co Fundación Universitaria Juan N Corpas Sistemas Fundación Universitaria Juan N Corpas Bogotá Colombia jenny.pinzon@juanncorpas.edu.co

**Keywords:** Trastornos de alimentación y de la ingestión de alimentos, adolescente, familia, ideación suicida *(fuente: DeCS, BIREME)*, Feeding and eating disorders, adolescent, family, suicidal ideation *(source: MeSH, NLM)*

## Abstract

**Objetivo:**

Estimar la relación entre el riesgo de trastornos alimentarios, la familia y la ideación suicida en una población adolescente femenina.

**Métodos:**

Estudio descriptivo, transversal. A lo largo de 12 años se aplicaron cuestionarios a 14 193 adolescentes femeninas escolarizadas de los colegios públicos de la localidad de Suba en Bogotá, seleccionadas voluntariamente. Como variables explicativas se indagaron el género, la edad, el estrato, la funcionalidad (usando el Apgar familiar), la estructura familiar (miembros con los que se convive), la ideación suicida, el estado de ánimo, la relación con los pares y la autoestima. Como variable dependiente se realizó el cuestionario Scoff. Se obtuvieron los OR de esta relación.

**Resultados:**

La edad media de la población fue de 13,9 años. Se encontró una pre-valencia de riesgo de trastornos de la conducta alimentaria de 28,4% y de disfunción familiar de 33%. La disfunción familiar severa aumentó la prevalencia de trastornos de la conducta alimentaria (OR 2,6, IC 95% 2,2-3,0) y de ideación suicida (OR 3,5, IC 95% 3,0-4,0).

**Conclusiones:**

Esta investigación confirma la importancia de la funcionalidad familiar en relación con el riesgo de trastornos de la conducta alimentaria, la ideación suicida y la distorsión de la imagen corporal. Con base en los hallazgos se propone implementar talleres que disminuyan los factores de riesgo causantes de los trastornos alimentarios y afiancen los factores protectores.

## Abstract (en)

**Objective:**

To show relationship among risk for eating disorders, suicidal ideation and family among female adolescents.

**Methods:**

Descriptive cross-sectional study. Over 12 years, questionnaires were applied to 14,193 female adolescents enrolled in public schools in Suba locality in Bogotá, they were selected voluntarily. As explanatory variables, gender, age, stratum, functionality (using the family Apgar) and family structure (members with whom they live), suicidal ideation, mood, relationship with peers and self-esteem were investigated. The ORs were obtained. The Scoff questionnaire was used as a dependent variable.

**Results:**

The mean age of the population was 13.9 years old. A prevalence of risk for eating disorders of 28.4% and of family dysfunction of 33% were found, severe family dysfunction increased the prevalence of eating disorders (OR 2,6, CI 95% 2.2-3.0) suicidal ideation (OR 3.5, CI 95% 3.0-4.0).

**Conclusions:**

This research confirms the importance of the family in relation to the risk for eating disorders, as well as the relationship among suicidal ideation and distortion of body image. According to the findings, it is proposed to implement workshops that reduce the risk factors that cause disorders and strengthen the protective factors.

Los trastornos de conducta alimentaria (TCA) se definen como una alteración persistente en la alimentación o en el comportamiento que conduce a un deterioro de la salud física o del funcionamiento psicosocial, lo cual genera una preocupación por el peso y el temor a engordar 1. La Organización Mundial de la Salud (OMS) ha ubicado a los TCA entre las enfermedades mentales de prioridad para los niños y los adolescentes. Estos trastornos representan la tercera enfermedad crónica más común entre jóvenes, con una incidencia del 5%, y son más prevalentes en las mujeres que en los hombres, con una relación 10:1 2.

El adolescente se encuentra en etapa de formación de su personalidad, vive un periodo de maduración psicológica y sufre por los cambios corporales, donde juzga y decide para insertarse en la sociedad 3, lo que podría provocar que incurra en conductas desadaptativas, como aquellas que alteran los hábitos alimentarios 4. La figura desempeña un papel importante en el desarrollo de la autoestima y por ello el control de peso se vuelve relevante con la finalidad de cumplir los estereotipos impuestos por la sociedad 4.

Sander 5 determinó que los adolescentes y los jóvenes, principalmente mujeres, son muy vulnerables al desarrollo de trastornos alimentarios, de manera más notoria entre los 14 y los 19 años 5,6. Muñoz 7 afirma que en Colombia estas patologías afectan especialmente a la población femenina joven, con un alto grado de incidencia en grandes capitales como Bogotá (12%) y Medellín (18%) en mujeres entre los 12 y los 21 años.

Se ha descrito que los TCA se asocian a las interacciones familiares 8, siendo el funcionamiento familiar uno de los factores implicados en la evaluación de la imagen corporal negativa en los pacientes con TCA, lo que refleja la correlación entre la toma de decisiones, la adaptabilidad y la cohesión 9. Ruiz 10 destaca que el funcionamiento familiar deteriorado es un aspecto consistentemente asociado con los TCA, y que se caracteriza por menos comunicación, dificultad en la solución de problemas y por compartir pocas actividades sociales, intelectuales y morales, además de presentar reglas límites estrictas 10.

Mateos 11 propone unas diferencias en las expresiones de la disfunción familiar de acuerdo al tipo de alteración alimentaria en los adolescentes, relacionando a padres que se entrometen mucho de forma no empática en el aspecto del cuerpo de los jóvenes, con la anorexia nerviosa y, en cambio, con la bulimia nerviosa, a padres distantes y conflictivos que no se acercan apropiadamente a los adolescentes.

Se ha detectado una relación entre disfunción familiar y riesgo de ideación suicida 12 cuando se sobrepasan los recursos adaptativos del joven, como lo son los mecanismos de defensa y la capacidad de apoyo y ajuste del entorno 13, de manera que el suicidio aparece como única solución para salir del impase y del estado de tensión interna en el que se encuentra.

En Colombia, el Instituto Nacional de Medicina Legal y Ciencias Forenses identificó una tasa de suicidio de 9,9% en la etapa de la adolescencia, comprendida entre los 12 y los 17 años 14. En relación con los pacientes con TCA, Martínez 14, en un estudio con población escolar de Boyacá, identificó que el 30,5% de los jóvenes en riesgo afirmó haber intentado suicidarse, en tanto el 42,4% de las estudiantes con trastorno alimentario presentó ideación suicida 15.

Thompson 16 observó que el grupo de pares puede influir en el desarrollo de TCA, mediante comentarios negativos, burlas y bromas, que los expone a desarrollar conductas alimentarias desadaptativas 16.

Esta investigación pretende estimar la relación entre el riesgo de TCA, la funcionalidad familiar y la ideación suicida en adolescentes femeninas escolarizadas de Bogotá, a lo largo del tiempo, buscando identificar características de la población en riesgo para implementar estrategias de promoción y prevención.

## MATERIALES Y MÉTODOS

### Tipo de estudio

Estudio observacional descriptivo de corte transversal. 

### Población

Adolescentes de sexo femenino escolarizadas con entre los 10 y los 20 años, seleccionadas voluntariamente durante los años 2006 a 2018 en colegios de la localidad de Suba (Bogotá).

### Los criterios de inclusión

Esta investigación forma parte del Programa Integral de Promoción de Salud del Adolescente (Pipsa), que se viene desarrollando desde el 2005 en la localidad de Suba por parte de la Facultad de Medicina de la Fundación Universitaria Juan N Corpas 17. A tal efecto, se invitó a participar a 12 colegios públicos de la localidad mediante las orientadoras. Aceptado el estudio por las directivas del colegio se acudió a los salones de secundaria y se explicó la investigación, así como la libertad para participar en ella y el manejo confidencial del cuestionario. El criterio de inclusión fue el pertenecer al colegio, estar en alguno de los cursos y querer participar en el estudio.

### Los criterios de exclusión

Sexo masculino, adolescentes que no hayan diligenciado de manera correcta y completa la encuesta.

### Variables

Como variables independientes se escogieron las características sociodemográficas (edad, sexo, estrato social medido en escala de 1 a 6, siendo 1 el más bajo); el estado de ánimo y la funcionalidad familiar, usando el test de Apgar original, valorando con 2 puntos las respuestas si suceden casi siempre; 1 punto si ocurren en algunas ocasiones y 0 puntos si prácticamente nunca suceden, con un rango entre 0 y 10 puntos 18. Los puntos de corte se sitúan entre 7 y 10 (normofuncional), 3 y 6 (disfuncional leve) y 0 y 2 (disfuncional grave). Para riesgo de suicidio se indagó por presencia de ideas suicidas (pensar y planear el suicidio). También se indagó por autoestima (creer que alcanzará lo que quiere, percepción de la imagen corporal y forma de ser) y, por una manifestación de *bullying* (los compañeros dicen que es torpe). Como variable dependiente se realizó el cuestionario Scoff, que consta de cinco preguntas que abordan el núcleo fundamental de la bulimia y la anorexia. Este instrumento se utiliza para establecer el riesgo de TCA. Las puntuaciones de 0-1 indican ausencia de trastorno alimenticio, en tanto que de 2 en adelante identifican riesgo 15.

### Recolección de la información

Las encuestas se realizaron entre los años 2006 y 2018 en los colegios públicos de la localidad de Suba (Bogotá), con la colaboración de estudiantes de la Facultad de Medicina de la Fundación Universitaria Juan N Corpas. Cada encuesta autodiligenciada tomó en promedio 20 minutos. Para llegar a los colegios se capacitó a un grupo de estudiantes de la facultad. Al llegar al salón donde estaban los jóvenes, se solicitó a los maestros que salieran, y se entregaban los formularios. Se ofreció un programa de consulta para los jóvenes que al llenar la encuesta percibiesen que deseaban ser asistidos médicamente. Cada colegio participante recibió el informe con el diagnóstico de riesgos.

### Manejo estadístico

Una vez se recibió la encuesta, se tabuló en el programa Excel y se construyó una base de datos para cada colegio. Se identificó a la población en riesgo de TCA y sus prevalencias en las variables independientes, las cuales fueron sometidas al proceso de análisis con Epi Info, y se obtuvieron los OR con sus límites de confianza y la prueba p respectivos.

## RESULTADOS

La Tabla 1 permite intuir la dinámica demográfica del paso de los años en los colegios públicos de la localidad.

**Tabla 1:** Características sociodemográficas de adolescentes escolarizadas en Bogotá, 2006-2018

Población	2006		2009		2010		2011		2013		2015		2016		2018	
	N°	%	N°	%	N°	%	N°	%	N°	%	N°	%	N°	%	N°	%
Edades																
10-12 años	555	24	67	29	106	22	21	24	62	34	19	20	104	25	62	26
13-16 años	1 343	33	199	34	390	28	70	28	111	31	88	27	406	35	196	29
17-20 años	144	32	45	38	69	38	8	16	8	30	14	23	54	32	50	39
Sexo																
Femenino	2 067	30	317	33	568	28	99	26	182	32	123	25	570	33	310	29
Estrato																
1 y 2	1 618	30	215	33	270	25	32	19	113	32	13	27	344	33	189	29
3 y 4	359	28	83	35	273	31	62	30	53	33	101	26	193	34	104	30
5 y 6	3	75	1	20	3	25	2	50	0	0	2	17	15	31	2	25

La Tabla 2 muestra las preguntas hechas a las adolescentes encuestadas para valorar la relación entre las variables y el riesgo de presentar TCA.

**Tabla 2:** Prevalencias de estructura y funcionalidad familiar, percepción del estado de ánimo y riesgo de depresión, suicidio, autoestima y trastornos de la conducta alimentaria en adolescentes escolarizadas en Bogotá, 2006-2018

Variable	N.°	%
Estado de ánimo		
Triste	1 339	9,5
Feliz	4 549	32,3
Normal	8 199	58,2
Funcionalidad familiar		
Disfunción severa	866	6,5
Disfunción moderada	3 831	28,7
Funcionalidad normal	8 654	64,8
Estructura familiar		
Hogar nuclear (vive con papá, mamá y hermanos)	6 812	49,5
Hogar incompleto (falta uno de los padres)	4 329	31,5
Hogar extenso (padres, hermanos y otros)	1 481	10,8
Hogar reconstituido (padrastro y madrastra)	944	6,9
Hogar sin padres	198	1,4
Riesgos de depresión, suicidio y autoestima		
Ha pensado en el suicidio, pero no lo haría	5 203	37,8
Lo ha pensado y le gustaría hacerlo	704	5,1
Ha buscado ayuda por haberlo pensado	1 358	10,2
Cree que en futuro alcanzará lo que quiere	10 553	76,8
No cree que alcanzará lo que quiere	458	3,3
No se imagina como será su futuro	2 732	19,9
Satisfecho con aspecto y forma de ser	8 464	63,7
Más o menos satisfecho	3 552	26,7
Poco o nada satisfecho	1 277	9,6
Le gustaría cambiar muchas partes de su cuerpo	715	5,4
Algunas partes	2 497	18,7
Pocas partes	3 225	24,2
No cambiaría nada	6 898	51,7
Los compañeros dicen que usted es torpe		
Muchos lo dicen	416	3,1
Algunos lo dicen	1 019	7,7
Muy pocos lo dicen	2 681	20,2
Ninguno lo dice	9 179	69,0
Riesgos de trastorno de conducta alimentaria		
Riesgo de trastorno de conducta alimentaria	4 236	29,8
Se provoca el vómito después de comer	1 597	11,9
Le preocupa haber perdido control en forma de comer	5 431	40,6
Ha perdido más de 7 kilos en los últimos tres meses	1 357	10,3
Cree que está gordo(a) así los demás le digan que está delgado(a)	3 947	29,5

La Tabla 3 muestra que cambiar muchas partes de su cuerpo es la relación más fuerte con el riesgo de TCA, seguida de pensar en el suicidio. La estructura familiar, con excepción de un hogar reconstituido, y los estratos socioeconómicos no supusieron ningún riesgo.

**Tabla 3:** Relaciones entre estado de ánimo, función familiar, ideas de suicidio, autoestima, relación con pares y tca en adolescentes escolarizadas, Bogotá 2006-2018

Variable	Sí TCA		No TCA		OR	IC 95%		Valor p
	N.°	%	N.°	%	LI	LS
Ánimo								
Triste	608	45,4	731	54,6	2,4	2,1	2,7	0,00
Feliz	1 174	25,8	3375	74,2				
Función familiar								
Disfunción severa	400	46,2	466	53,8	2,6	2,2	2,9	0,00
Funcionalidad normal	2 163	25	6 491	75				
Disfunción moderada	1 425	37,2	2 406	62,8	1,8	1,6	1,9	0,00
Funcionalidad normal	2 163	25	6 491	75				
Disfunción severa	400	46,2	466	53,8	1,4	1,2	1,6	0,00
Disfunción moderada	1 425	37,2	2 406	62,8				
Ideas de suicidio								
Lo ha pensado y le gustaría hacerlo	353	50,1	351	49,9	3,5	2,9	4,0	0,00
No lo ha pensado	1 764	22,5	6 082	77,5				
Ha pensado en el suicidio, pero no lo haría	2 051	39,4	3 152	60,6	2,2	2,1	3,1	0,00
No lo ha pensado	1 764	22,5	6 082	77,5				
Autoestima								
¿Cree que en futuro alcanzará lo que quiere?								
No cree que alcanzará lo que quiere	237	51,7	221	48,3	2,6	2,1	3,1	0,00
Cree que en futuro alcanzará lo que quiere	3 099	29,4	7 454	70,6				
¿Está satisfecho con su aspecto y forma de ser?								
Poco o nada satisfecho	638	50	639	50	2,9	2,6	3,3	0,00
Satisfecho con aspecto y forma de ser	2 157	25,5	6 307	74,5				
¿Le gustaría cambiar muchas partes de su cuerpo?								
Le gustaría cambiar	476	66,6	239	33,4	7,3	6,2	8,7	0,00
No cambiaría nada	1 474	21,4	5 424	78,6				
Relación con los pares								
Los compañeros dicen que usted es torpe								
Muchos lo dicen	230	55,3	186	44,7	3,3	2,7	4,0	0,00
Ninguno lo dice	2 475	27	6 704	73				

**Figura 1:**
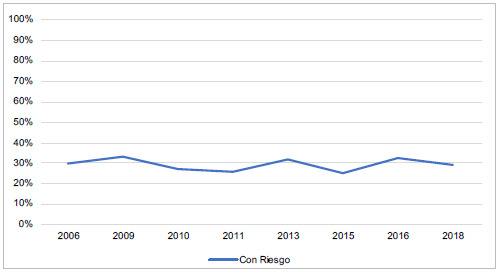
Tendencia de riesgo de TCA del 2006-2018

La Figura 1 muestra cómo a lo largo de los años la tendencia del riesgo de trastornos del comportamiento alimentario se mantiene en un 30%, aproximadamente, no se evidenció variación estadística de un periodo a otro con el paso de los años.

## DISCUSIÓN

Los sesgos de información a los que se expone la presente investigación están sujetos a la limitación propia de las encuestas, a saber: que no se conteste con la verdad. Sin embargo, tal limitación se enfrentó mediante la similitud de los resultados obtenidos a lo largo de los años, el cumplimiento del anonimato de cada formulario, el manejo de la confidencialidad de la encuesta, la insistencia en la garantía de la voluntariedad y la exclusión de profesores en el momento de realizar la encuesta. Además, los resultados obtenidos guardan correspondencia con las referencias consultadas, lo que hace suponer que los sesgos se superaron suficientemente como para darle validez al estudio, teniendo en cuenta que este trabajo fue realizado por conveniencia.

Encontramos una prevalencia de riesgo para el desarrollo de riesgo de TCA en un 30%, aproximadamente, sin variación significativa a través del tiempo y con mayor incidencia entre las edades de 17 y 20 años (Figura 1). Datos similares a los de Fajardo 19, que identificó un 30% de riesgo de presentar TCA en población de escolaridad secundaria y, un poco mayor al encontrado por Manrique 20, con un 26,5 % en adolescentes entre los 16 y los 17 años.

El estrato socioeconómico no hace diferencia con respecto al riesgo de TCA. Hallazgo similar al de Ángel 21, lo que expone de algún modo la universalidad del problema.

En el presente estudio se encontró que jóvenes que se perciben tristes tienen una relación con el riesgo de TCA (Tabla 3), contrariamente a lo que afirma Peña 22. Esta diferencia se podría explicar por la relación aquí encontrada con querer cambiar muchas partes del cuerpo y no poder hacerlo (Tabla 3).

Esta investigación (Tabla 3) ratifica la relación entre riesgo de TCA y disfunción familiar, similar a lo encontrado por otros investigadores 9,10,23-25. Una explicación puede ser que la disfunción familiar se expresa por comunicaciones negativas en torno a la forma de comer de alguno de los miembros hacia la adolescente. También el exceso de control, la sobreprotección, así como el impedir su independencia, causa en los adolescentes la impresión de que no tienen el control de sus vidas, les hace pensar que en lo único que pueden ejercer sus decisiones es en su propio cuerpo 23, apoyando así los resultados anteriormente mencionados en la presente investigación. Reitera esta investigación que es como funcione la familia y no la cantidad de miembros lo más importante 23 en torno a los riesgos de TCA.

Este estudio confirma las relaciones entre ideación suicida y riesgo de TCA (Tabla 3), similar a lo encontrado por Duffy 13. Si los riesgos de TCA constituyen un indicador que podría indicar a su vez a un riesgo de suicidio en la población adolescente 13, identificarlos prontamente puede servir para prevenir este último.

La imagen corporal (Tabla 3) permitió evaluar en este estudio la significancia estadística entre las adolescentes con riesgo de TCA y el hecho de querer cambiar muchas partes de su cuerpo, confirmando lo descrito por Perkins 26 y proponiendo que puede ser otro predictor significativo de pensamientos suicidas.

Se encontró una relación significativa entre el riesgo de TCA y ser víctima de bullying (que los compañeros le digan torpe, Tabla 3), similar a lo encontrado por Copeland 27, quien identificó mayor riesgo de desórdenes alimentarios, percepciones negativas del propio cuerpo, efectos sobre la autoestima y problemas emocionales en las víctimas de *bullying.* Asimismo, Lee 28 muestra que el acoso por parte de compañeros puede conducir a que las adolescentes presenten síntomas depresivos, aumentando con ello el riesgo de conductas alimentarias desordenadas. Si vemos lo relacionado (Tabla 3), existe una asociación entre disfunción familiar, ideación suicida, *bullying* y riesgos de trastornos de conducta alimentaria (Tabla 3), con altas prevalencias (Tabla 2) que ponen de manifiesto la necesidad de intervenir a nivel de los colegios 29.

En conclusión, este estudio demuestra que existe suficiente evidencia de relaciones y explicaciones entre los riesgos de TCA y la falta de aprecio por imagen corporal, disfunción familiar, ideación suicida y *bullying* y que se presenta desde el comienzo de la adolescencia. Dichas relaciones pueden expresar sufrimiento emocional, haciendo necesario proponer intervenciones de manejo a las emociones, autoimagen, comunicación interpersonal, así como estilo de vida y alimentación adecuada, a nivel de los colegios 21,30. Además, si el propósito es impulsar la atención primaria en salud, los equipos de atención 31 deben ser debidamente preparados para identificar a la población de riesgo e intervenirla preventivamente, incluyendo a la familia 32,33.
